# Photonic Crystal Enhanced Fluorescence: A Review on Design Strategies and Applications

**DOI:** 10.3390/mi14030668

**Published:** 2023-03-17

**Authors:** Yanyu Xiong, Skye Shepherd, Joseph Tibbs, Amanda Bacon, Weinan Liu, Lucas D. Akin, Takhmina Ayupova, Seemesh Bhaskar, Brian T. Cunningham

**Affiliations:** 1 Department of Electrical and Computer Engineering, University of Illinois at Urbana-Champaign, Urbana, IL 61801, USA; 2 Nick Holonyak Jr. Micro and Nanotechnology Laboratory, Urbana, IL 61801, USA; 3 Department of Bioengineering, University of Illinois at Urbana-Champaign, Urbana, IL 61801, USA; 4 Department of Chemistry, University of Illinois at Urbana-Champaign, Urbana, IL 61801, USA; 5 Carl R. Woese Institute for Genomic Biology, Urbana, IL 61801, USA; 6 Cancer Center at Illinois, Urbana, IL 61801, USA

**Keywords:** photonic crystals, fluorescence, plasmonic, nanoengineering, photonics, fibers, opals, biosensing, defect, cavity, Bloch surface waves, diagnostics

## Abstract

Nanoscale fluorescence emitters are efficient for measuring biomolecular interactions, but their utility for applications requiring single-unit observations is constrained by the need for large numerical aperture objectives, fluorescence intermittency, and poor photon collection efficiency resulting from omnidirectional emission. Photonic crystal (PC) structures hold promise to address the aforementioned challenges in fluorescence enhancement. In this review, we provide a broad overview of PCs by explaining their structures, design strategies, fabrication techniques, and sensing principles. Furthermore, we discuss recent applications of PC-enhanced fluorescence-based biosensors incorporated with emerging technologies, including nucleic acids sensing, protein detection, and steroid monitoring. Finally, we discuss current challenges associated with PC-enhanced fluorescence and provide an outlook for fluorescence enhancement with photonic-plasmonics coupling and their promise for point-of-care biosensing as well monitoring analytes of biological and environmental relevance. The review presents the transdisciplinary applications of PCs in the broad arena of fluorescence spectroscopy with broad applications in photo-plasmonics, life science research, materials chemistry, cancer diagnostics, and internet of things.

## 1. Introduction

The pigments and bioluminescence of natural species employ advanced modes of light–matter interactions and suggest concomitant directions for scientific research. Interestingly, certain species, including butterflies, birds, and fish, display magnificent color patterns even in the absence of pigments or intrinsic luminescence [1,2,3,4,5]. The unique interaction of light with the nanoscale, layered structures of these species enables such structural coloration on account of interference phenomena [6,7,8]. These observations have inspired scientists to understand and mimic structurally layered patterns of different refractive indices comprising micro-nano systems to demonstrate photon/light modulation with engineered structures. A variety of engineering methodologies have been explored in order to confine, control, collimate, direct and configure the optical energy with both passive nanostructures and integrated optoelectronic devices. Among them, photonic crystals (PCs) and associated technologies have offered flexible and versatile routes to manipulate light energy at micro-nanoscale dimensions [6,9,10,11]. 

The light–matter interactions associated with PCs are defined by the periodic arrangement of the materials with different refractive indices (or dielectric constants). Since their inception through the foundational reports by Yablonovitch [12] and John [13] in 1987, the study of such regular/periodic arrangements of materials has been utilized for achieving controlled propagation of photons, in the same way as the electron motion is manipulated in energy bands (allowed and forbidden) in semiconductor crystals. Hence, structures that allow the manipulation of photon propagation by virtue of their periodically monitored internal domains of high and low dielectric constants are referred to as PCs. Since the periodicity in the PCs is at the length scale that matches the wavelength of light under consideration, it provides opportunity to tailor the optical field propagation [7,10,11,14].

In the past decade, many publications have elaborated on the fundamental theory, physics equations, and associated design principles of PCs [2,6,9,10,11,14,15]. Depending on the periodic modulation of the refractive indices in the materials constituting the PCs in the spatial domain, one-dimensional (1D), 2D, and 3D PCs have been designed, simulated, fabricated, and experimentally characterized. While PCs are defined by structural properties described above, a functional hallmark of many PCs is the phenomena of a photonic band gap (PBG) that is extensively explored in different research disciplines [7,10,15,16]. In other words, it is important to note that every PC need not generate a PBG, whereas every structure that sustains a PBG is categorized as a PC. The PBG is the region of the electromagnetic spectrum of specific frequencies that are not allowed to pass through the PC and are directly reflected to the surrounding medium. Such back-reflection occurring at visible wavelengths results in brilliant colors of PC surfaces resembling the previously mentioned color patterns in butterflies achieved through interference of light (comprising reflections and refractions of photons) with the PC’s high and low refractive index regions, thereby generating constructive and destructive interference patterns. In addition, the fundamental physics formulas and equations pertaining to eigenfunctions (or resonant wavelengths) of PCs can be extrapolated and adopted to simulate, and strategically design PCs presenting PBG at desired regions of the EM spectrum [11,14,15,17,18].

Recently, the light guiding properties of PCs have been leveraged to accomplish desired optical functionalities and is found in applications encompassing (but not limited to) light harvesting, biosensors, analytical tools, information encryption, photodynamic antibacterial study, display technologies, drug delivery, nano-resonators, lasers, anticancer therapy, green printing, photocatalysis and the internet of things (IoT) [9,10,14,19,20,21,22,23,24,25]. The unique properties of PCs make them attractive for various integrated-device applications being developed into commercial products, notwithstanding certain challenges of manufacturability and intrinsic defects observed in 3D PCs [26,27,28,29,30,31,32]. Among several such interdisciplinary applications of PCs, the capability of the PCs to drastically alter the light-emitting characteristics of adjacently located luminescent species has gained substantial attention from the research community at the crossroads of physics, chemistry, materials science, and life sciences [16,26,27,28,29,30]. Such explorations of investigating the interaction of the phenomena supported by PCs (such as a cavity, band edge, and internal modes) with light-emitting reporters, particularly fluorophores, used in biodetection have generated intriguing results, demanding a comprehensive outlook [33,34,35]. Encouraged by this understanding and from the authors’ earlier contributions in the field [15,20,36,37,38,39,40,41,42,43,44], through this focused review, we make an attempt to discuss the recent developments in PC-enhanced fluorescence and related modalities.

Fluorescence spectroscopy and associated applications are emerging as a central aspect of technologies for environmental (soil, air, and water) and biological (aquatic and terrestrial lifeforms) health monitoring [45,46,47,48,49,50]. The biosensing of ions, molecules, analytes, toxins, and biomarkers relevant to point-of-care (POC) diagnostics has seen a paradigm shift in terms of sensitivity on account of the high quantum yield rendered by novel photon emitter systems. Chemists have developed new molecules and complexes with intrinsically high quantum yield coupled with the ability to selectively interact with the target analytes. To complement these advances, it is now firmly established that emission from fluorophores can be drastically modulated by placing them in the proximity of nanostructures that significantly alter their local density of states (LDoS) and photostability in the near-field [48,51,52,53,54]. In order to accomplish this, a variety of micro and nano-systems have been demonstrated [55,56,57,58,59]. In recent years, the research and development pertaining to the interaction of fluorescent moieties with plasmonic/metallic nanoparticles have exponentially improved with novel applications [60,61,62,63,64,65,66,67,68]. However, the high Ohmic losses and quenching effects encountered in plasmonic nanomaterials have remained a major bottleneck to realize unprecedented enhancements in fluorescence [15,46,69,70,71,72,73,74]. On account of such high energy dissipation pathways observed with metal-dependent plasmonics, the focus in the past two decades has shifted towards the implementation of lossless dielectric platforms for fluorescence enhancement applications [62,63,64,65,66,67,68]. Consequently, modulation in the radiative, non-radiative decay pathways and the associated lifetimes and quantum yields have been achieved. Such hybrid synergistic approaches have assisted in tuning the light–matter interaction between the emission from the fluorophores and the PCs in a wide region of the EM spectrum for applications from infrared (IR) to ultraviolet (UV) light, enabled by optical properties of PCs [75,76,77,78,79,80].

In this background, myriad spectroscopic applications stemming from the interaction of PCs with fluorophores exist. The relative position of the fluorescence excitation and emission maximum, as well as the optical resonances contributed by the interacting PCs substantially influences the nature of the combined platform [78,79,81]. Enhanced excitation and extraction of emission have been achieved in different fluorophore–PC ensembles with the careful design of guided mode and leaky mode resonances [33,82]. Improving the fluorescence signal intensity is an indispensable goal of biosensing approaches, as it renders augmented sensitivity [83,84,85,86,87,88]. In light of these perspectives, through this focused review, we aim to summarize such explorations of fluorophore–PC ensemble by rationally categorizing the topics based on the types of PCs and their resonance properties in fluorescence-based applications.

In this review, we begin by presenting an overview focusing on the fundamentals of fluorescence and PCs. Next, recent developments in employing novel PC platforms designed through opal and inverse opal configurations, and PC fibers for fluorescence-based applications are discussed. In addition, the effect of certain optical functionalities, including the defect, band edge, and cavity modes sustained by the PCs for effectiveness in modulating the fluorescence signal intensity is summarized. The aforementioned aspects are briefly deliberated to provide a glimpse of the large landscape of opportunities available for tailoring the fluorescence. Further, a more thorough review is presented in the scenarios pertaining to photonic crystal-coupled emission (PCCE) and photonic crystal enhanced fluorescence (PCEF). We have carefully chosen these platforms on account of their contemporary relevance and cutting-edge research findings, that has broadened the horizon of understanding the coupling between the fluorophores and the PCs [41,78]. In these systems, innovation is driven by the understanding of optical field coupling between the emitted photons and the modes sustained by the PCs, inextricably linking the developments of chemical and engineering aspects. Although the peculiarities of these platforms yield distinct outputs for biosensing applications, they substantially differ in structural design and consequential photonic response [41,78]. On the one hand, the PCCE platform is equipped with the ability to exploit the utility of surface electromagnetic waves termed Bloch Surface Waves (BSWs) and internal optical modes with a prism-dependent technique [39,41,43,56]. On the other hand, the PCEF platform presents an elegant framework to utilize the guided modes in surface patterned Bragg grating to optically couple with the photons emitted by the proximal fluorophores via a simplistic and prism-independent approach [37,77,78,79].

While the reader interested in gaining a deeper understanding of the theory and applications of PCs in various multi-disciplinary domains is encouraged to explore published books and reviews [6,9,10,21,22,23,24,25,89,90], this review focusses on presenting recent developments in the research pertaining to the combination of fluorescence applications in specific PC frameworks. In the following sections, the recent developments in the broad arena of PCs and their applications in fluorescence enhancements is discussed. It is our goal for this focused review to provide an overview of the latest advancements in fluorescence enhancements using PCs, thereby guiding scientists researching in the intersection of physics, chemistry, biosciences, and medical diagnostics. We conclude by sharing prospects and opportunities for future research in this direction, thereby fostering transdisciplinary applications at the confluence of the photonics and luminescence research community.

## 2. Synergizing Fluorescence and PCs

### 2.1. A Brief Overview of Fluorescence and Related Processes

Fluorescence is a rapid process of nanosecond-scale spontaneous emission, where mitted photons from fluorophore undergo electronic transition from excited states to ground state after a vibrational relaxation. There are several mechanisms to cause electron exists at excited state (relatively energy unstable), in particular, absorption of radiation (excitation with shorter wavelength), mechanical action (mechanoluminescence), highly exothermic chemical reactions (chemiluminescence), radiative recombination of electrons and holes (electroluminescence), or even sound (sonoluminescence). In this review, we are only focusing on light excitation which is more commonly used in biosensors. Fluorescent moieties are used within flow cytometers, microscopes, lab-on-a-chip, and diagnostic devices due to fluorescent dye’s versatility in detection instruments. Fluorescence biosensors are widely used despite work on efficient light extraction still developing. However, only a small fraction of the emitted light is typically detected (<30%) even with sophisticate optical set-ups while require a high-power excitation source [55,91]. A sensor substrate that can enhance excitation, extraction and quantum efficiency is urgently needed for fluorescent-based detection. 

### 2.2. A Brief Overview of PCs

Since their introduction, PCs have been extensively explored to take advantage of their ability to control light. PCs are generally made of periodic arrangements of high and low refractive index materials. These periodic arrangements can be made in multiple dimensions where properties are manipulated largely through refractive index contrast and arrangements of elements [92,93]. A variety of geometries, including Bragg reflectors, slabs, opals, microcavities, and colloids, can be used to construct PC structures. One-dimensional PCs, with one direction of periodicity, also known as Bragg stacks/reflectors, are used in antireflective coatings on glass and other optical surfaces [38,94,95].

**Figure 1:**
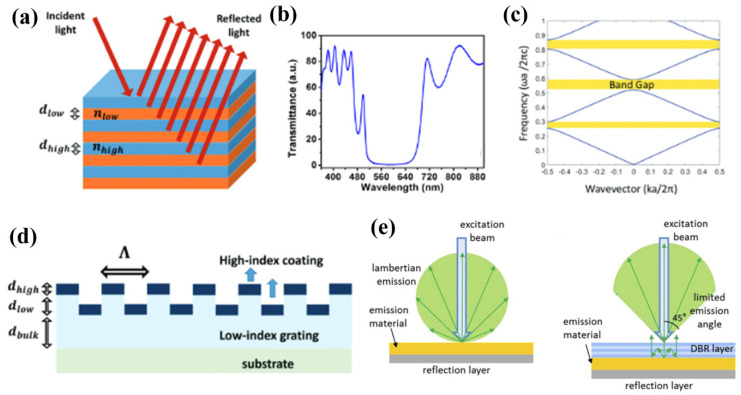
(**a**) A Bragg reflector showing the increased total reflection due to consecutive layers of high and low refractive index, adapted with permission from [38]. (**b**) Transmittance spectrum of a Bragg reflector, adapted with permission from [43]. (**c**) Example of band gap structure of a 1D PC. (**d**) Example structure of 1D PC slab with an alternating grating of high and low reflective index, adapted with permission from [38]. (**e**) Example emission of the guided emission from a distributed Bragg reflector (DBR) versus a non-optically active material, adapted with permission from [95].

PC slabs are usually fabricated by a highly refractive layer periodically layered over a lower refractive index layer such that the period of the grating is less than the wavelength. Similar to the bandgaps of electrons in solids, the bandgap of photonic crystals can be interpreted as the destructive interference of many reflections of light traveling in the crystal at each interface between layers of high- and low-index regions, as seen in Figure 1a–d. These slabs are often structured such that the subwavelength grating (period of the grating is smaller than the wavelength of light) produces optical resonances. They also produce sharp peaks in the reflection spectrum as only a narrow band of wavelengths will be reflected. Under certain exciting conditions, PCs can support guided mode resonances that can channel internal light externally [82]. Such processes have been exploited to increase the emission collection efficiency, as demonstrated in Figure 1e [96,97,98].

n λ  =  2d sinθ (1)

For most PC structures, Bragg’s law can be used to describe the phase-matching of coupling conditions, where n is the diffraction order, λ is the wavelength, d is the distance between layers of atoms in a crystalline solid, and θ is the coupling angle as seen in Equation (1). 

Two-dimensional PCs have periodicity in two spatial dimensions and thus can be manipulated for properties such as diffraction and reflection enhancement [99,100]. Three-dimensional PCs properties focus on efficient diffraction and lasing [101,102]. Photonic slabs, patterned 1D and 2D slabs of PCs, are especially appealing for microfluidic biosensing due to their good sensitivity, simplified optics, and compatibility with substrates [103,104]. As semiconductors led to the integration of electronics, PCs are thought to enable optical integration especially with their ability to be created from any material as they are created from high index refraction to low refraction indexes. The 2D structures have been utilized extensively due to simple and manufacturable fabrication (further discussed in a review by Krauss and De La Rue [105]). Due to the guided resonances, with at specific combinations of incident angle and wavelength, PCs also have enhanced excitation effects over optically passive surfaces such as glass. At the resonant coupling condition, guided modes are excited within the PC, creating an evanescent field where fluorophores will receive greater illumination intensity than they would experience on an unpatterned surface. This augmented intensity is created without using a higher-powered source. Consequently, the effective use of PCs allows for less background noise (commonly associated with high-intensity power) with the use of inexpensive lower-power laser sources. 

Label-free optical sensors convert biological data to optical signals largely by detecting changes in the reflected or transmitted spectrum from the PC, where shifting refractive index results in measurable changes in intensity or wavelength, which in turn correlates with the presence and concentration of a biological analyte. By measuring changes in the sensor’s optical properties over time, biomolecular interactions on the surface of the PC can be detected through the intrinsic dielectric permittivity of the analyte molecules [18,106]. While label-free biosensing with PCs offers simple assay workflows, labeling of analytes with photon-emitted tags such as fluorophores offers routes toward reduced limits of detection. Therefore, in this review, we focus our discussion upon fluorescence-based applications using PCs [107].

## 3. Fluorescence-Based Applications: The Utility of Opals and Inverse Opal PCs

### 3.1. Opal and Inverse Opal PCs

PCs with three-dimensional periodicity are structurally subcategorized into two distinct groups: colloidal crystals (opals) and their reciprocal macroporous template lattices known as inverse opals. The periodicity associated with the specific dielectric constants of opal and inverse opal PCs determines the specific band of wavelengths whose propagation through their lattice structure is forbidden; known as the photonic band gap (PBG)–akin to the electronic band gaps observed in semiconductors materials–PBGs in PCs typically span a range of wavelengths within the ultraviolet (UV) and visible light spectral windows (10–350 nm and 350–750 nm, respectively). When the propagation of a specific range of wavelengths is forbidden in all directions and polarization phases across a crystalline lattice, it is said to exhibit a complete PBG, and a variety of opal and inverse opal PCs have been developed in recent years that exhibit such behavior over distinct optical regimes.

### 3.2. Fabrication of Opal and Inverse Opal PCs

**Figure 2:**
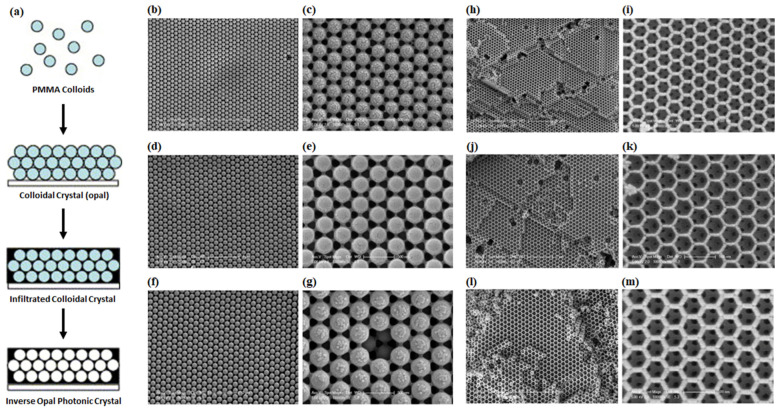
Overview of general fabrication strategy and structures of opal and inverse opal photonic crystals. (**a**) Schematic representation of the assembly process for fabrication of opal photonic crystals (OPCs) and inverse opal photonic crystals (IOPCs) from precursor PMMA colloidal microspheres. (**b**–**g**) SEM images of PMMA colloidal crystals fabricated using via flow-controlled vertical deposition. (**h**–**m**) SEM images showing SiO_2_ inverse opals made from colloidal crystals templates shown in sub-figures (**b**–**g**) (**left**). (Adapted with permission from [109]).

Colloidal crystals (opals) may be prepared by various methods falling under two distinct technical classifications: self-assembly or lithographic fabrication [108]. Self-assembly approaches—commonly referred to as “bottom-up” fabrication strategies—entail the crystallization of a colloidal suspension of preformed, monodisperse nanoparticles or microparticles, which are typically comprised of silica (SiOx), polymethylmethacrylate (PMMA), or polystyrene as illustrated in Figure 2 [108,109]. Although less precise by comparison to nanolithography-based approaches (“top-down” fabrication), self-assembly methods are often employed for routine applications, due to their synthetic flexibility, low cost, and ability to produce various pattern sizes (~10 nm–10 μm) over a large dimensional range (0.01–100 cm^2^) with thickness upwards of several hundred structural layers [109,110]. Since the rate of sedimentation influences the equilibrium configuration of colloidal assemblies, most practical applications require substrate deposition to proceed over relatively short durations. Considering the beforementioned criteria, various processes based on evaporation-driven convective assembly have been developed to control temperature and–by extension–the rate of particle-substrate deposition; included among this list of developed methodologies are sedimentation [111], vertical dip-coating [112], capillary infiltration [113], patterned growth epitaxy [114], and spin-coating [115]. Despite their utility and synthetic versatility, however, self-assembly methods are error-prone and commonly produce lattice structures with random defects [116,117], such as particle vacancies and colloidal dislocations with mixed phase alignments. Moreover, the breadth of lattice structures achieved via colloidal assembly is rather limited and typically restricted to the formation of structures with face-centered-cubic (fcc) and hexagonal close-packed (hcp) symmetries [109]. Owing to these limitations, various nanolithography-based approaches incorporating parallel optical processing have been developed in recent years—e.g., interference lithography (IL) [118], two-photon lithography (TPL) [119], and glancing angle deposition (GLD) [120]—enabling the rapid generation of highly periodic 3D structures with sub-micrometer periodicity that are nearly defect-free over large (cm^2^) areas. However, rather than utilizing monodisperse suspensions of nanoparticles to furnish opal- and inverse opal-type PC structures, these methods incorporate distinct source materials such as photosensitive polymers (IL), monomeric liquids and light-activated films (TPL), or metal oxides combined with generalized semiconductor templates (GLD) to 3D photonic structures.

Despite the continuous expansion of array fabrication approaches, including those employed for the assembly of 3D PCs suitable for applications in near-and far-IR spectroscopic analysis, few examples are available that illustrate a complete PBG (experimentally) for fabricated 3D opal photonic crystals (OPCs). This absence of a complete PBG in opaline crystals stems from the periodicity and volume-filling fraction occupied by the nanoparticles utilized during assembly, whose width modulates the optical contrast of the concomitant lattice structure and render a refractive index (n) below the minimum value required contrast value of 2.9 that is necessary to establish a discreet optical band gap. Conversely, several templating approaches have been investigated that utilize physiochemical backfilling approaches to infiltrate minimally refractive opal template structures with photo-processable nanoparticle emulsions [121], or polymerizable liquid suspensions [122], to furnish highly refractive inverse opals with complete PBGs specific to a user-desired optical regime.

### 3.3. Opal Photonic Crystals (OPCs) for Fluorescence-Based Applications

Not surprisingly, and like most imaging applications incorporating PCs, fluorescence-based approaches have sought to develop opal and inverse opal structures exhibiting precise and tunable PBGs; in light of this preferential condition, inverse opals have become the predominant template PC structure used for modern fluorescence imaging. However, various examples in recent years have incorporated opal PCs for fluorescence imaging and analysis [123] and assessed the fluorescence properties of modified coumarin (C6) dye structures dispersed in solution, as dye aggregates in organic phase media, and adapted to PMMA-based opals. In this work, the authors revealed that opal PCs effectively inhibited the aggregation-promoted quenching of various C6 dye conjugates through scission of intermolecular interactions between individual dye molecules, which they accomplished by manipulating the excitation wavelength and concomitant emission of the fluorophores through tuning of the PCs photonic stop band (PSB) and localization of the dyes within the microporous mesh present within the PMMA-based nanoparticles comprising the PC assembly.

A related work [124] studied the emission profiles of rhodamine 6G (R6G) dye molecules confined within the hexagonal closed-packing (hcp) lattice of opal PCs constructed through the self-assembly of colloidal polystyrene nanoparticles. In agreement with results obtained by Siu et al. for the C6 dye family, the authors confirmed that the embedded R6G fluorophores exhibited minimal variation in fluorescence lifetime as a function of the emission frequency within their emission spectrum. However, the authors determined that the fluorescence lifetime was a function of the crystal lattice parameter that agreed with theoretical calculations of the local density of optical states (LDOS) observed in the 3D periodic arrangements exhibited by opaline PCs. Taken together with theoretical calculations, these experiments revealed that the emission lifetime of confined fluorophores are modified over expanded frequency ranges compared to their standard emission profiles in free solution.

Towards the development of enhanced fluorescent dye reporters with complex emission profiles [125], Gao et al. developed a novel dual-mode PC with a multi-colored optical band profile. The authors incorporated UV-responsive colloidal spheres fabricated via polymerization of quasi-continuous emulsions endowed with fluorescent precursors functionalized with reactive carbamate moieties locally restricted to the shell of custom colloidal nanoparticles. Instead of solution-mediated condensation, UV irradiation was employed to initiate the photo decomposition of the carbamate groups in the fluorescent precursors and generate the concomitant amine handles as a byproduct. Through this approach, fluorescence generation was observed directly on the surface of 3D PC opals upon addition and reaction with the amine-specific reporter fluorescamine. In this work, the authors also established a method for constructing the two-dimensional (2D) multicolored photonic pattern equivalents of the parent 3D opals, thus enabling reciprocal imaging in either reflection- or fluorescence-specific formats.

### 3.4. Inverse Opal Photonic Crystals (IOPCs) for Fluorescence-Based Applications

IOPCs are optical micro-structures exhibiting periodic arrays of wave guide gratings capable of manipulating the propagation of light across their surface [126]. In most routine applications, IOPCs are constructed via self-assembly of colloidal nano- and micro-spheres. Although lithographic approaches have emerged that facilitate the controlled assembly of nearly defect-free IOPCs, a vast majority of these structures are fabricated by infiltration of filling materials in the 3D colloidal OPC template; several such backfilling procedures have been developed to access IOPCs, including sol-gel infiltration, atomic layer deposition (ALD), chemical vapor deposition (CVD) and electrochemical deposition. The most common infiltrating material utilized to assemble IO lattices include zinc oxide (ZnO), titanium oxide (TiO_2_), silicon dioxide (SiO_2_), mesoporous hydrogels, and carbon (graphite). A related review [127] provides in depth coverage of IOPCs templated from TiO_2_, which exhibit high refractive index (>2.5) yielding complete PBGs, notable biocompatibility, and exceptional solvent resistance. Generalized titanium oxide (TiOx) assemblies also exhibit photonic localization, delayed light effects, and negative refraction effects.

Expanding upon the molecular composition profile of IOPCs, Wang et al. developed a catalog of 3D inverse opals assembled from heterogeneous composite microspheres (HCMs) constructed from the noble oxides of indium and zinc (In_2_O_3_:ZnO) [128]. Motivated by the limiting humidity dependence and long recovery times of sensing elements in conventional room-temperature gas sensors, the authors developed a novel IOPC assembly approach premised on ultrasonic spray pyrolysis (USP) using self-assembled sulfonated polystyrene (S-PS) spheres as a sacrificial template. This novel assembly approach and utilization of hybrid metal oxide microspheres furnished highly ordered 3D inverse opal structures with meso- and macro-scale pore networks, which enhanced the sensitivity of gas detection through acceleration of the mass transfer rate (by virtue of the expanded accessible surface area of the opals molecular architecture). Furthermore, n–n heterojunctions established by the mixed oxide microspherical assembly extended the photo absorption window of the sensor to the visible light spectrum, which effectively prolonged the lifetime of photo-activated charge transfer. As prepared, their 3D IOPCs assembled by In_2_O_3_–ZnO HCMs enabled excellent sensitivity for the detection of NO_2_ under visible light irradiation at room temperature, achieving a lower detection limit of 250 ppb, sensor recovery time of approximately 3 min, excellent chemical selectivity and 100% humidity tolerance. Beyond their broad utility scope as primary detection elements in chemical sensors, solar cells, photocatalysts, optical fibers, and semiconductor heating elements, substantial research has focused on the biochemical modification of TiOx IOPCs for translation of these wave guiding elements into fluorescence-based imaging platforms (e.g., direct surface fluorescence, quantum dots, and FRET enhancing arrays).

As a direct extension of IOPCs for fluorescence-amplified chemical sensing, Li et al. developed a novel method for the detection of trace quantities of the explosive 2,4,6-trinitrotoluene (TNT) [129]. Compared to control samples, the fluorescence enhancement exhibited by their optimized PC assembly was greater than 60-fold above background in the presence of TNT, which was achieved as a result of the slow photon effects caused by the large intermolecular surface areas of the IOPC. Furthermore, the quenching efficiency of their novel chemical sensor was >80% after exposure to TNT vapor for a period of more than 300 s (>5 min).

In the area of biomedical engineering, integrated artificial fractal structures have garnered strong interest for the selective detection and capture of circulating tumor cells (CTCs) due to their known correlation with the diagnosis and prognostic implications of various types of cancer. In an effort to enhance the selective recognition and capture of CTCs in biological samples, Xu et al. designed a highly porous IOPC constructed from colloidal TiO_2_-Fe_3_O_4_:C_6_SiOx microparticles modified to display the anti-EpCAM antibody on their external interface via bioconjugation with polydopamine (PDA) as an intermediate linkage (PDA) [130]. The capture efficiency of this platform increased by more than 20-fold relative to the non-conjugated (control) IOPC assembly, and more than 200-fold compared to the capture efficiency achieved by poly-L-lysine coated flat substrates of equal dimensions. The authors noted that their novel IOPC substrate exhibited a controlled pore size of 415 nm which was responsible for the optimized immobilization and CTC capture efficiency (92%) using blood volumes administered across the IOPC chip at a rate of 1 mL/hr. Beyond the improved cell affinity, these IOPCs also exhibited enhanced scattering properties which enhanced the excitation and emission of fluorescent antigens bound to the conjugated antibodies found on the TiO_2_ microparticles, thus enabling this platform to be used for real-time monitoring of CTC capture.

In summary, it has been observed that in the past decade, the OPC and IOPC have been extensively explored for modulating the fluorescence signal intensity, thereby aiding the development of efficient biosensing platforms. The careful consideration of the photonic band structures (band gap and band edge) as well the absorption and emission maximum of the fluorescent moieties are the crucial for development of promising biosensing strategies, especially for substrates involving FRET phenomena and quantum dots. The influence of the underlying PC on the local density of states as well as the lifetime of the fluorophores provides significant insights in this regard. This is predominantly affected by the central position, band-width, and amplitude of the spectra as well as the relative dielectric constant of the ambient medium. From a futuristic scope point of view, these PC structures can be integrated to appropriate optical components realize angle-dependent or an angle-independent emission from the fluorophores embedded in the OPCs and IOPCs. Further, recently, there has been significant interest in the incorporation of biocompatible nano- and micromaterials in the fabrication of OPC and IOPC, so as to foster green nanotechnology. Although such attempts have demonstrated intriguing results, the experimental artifacts introduced during the fabrication process of substrates that are used for fluorescence-based ‘turn-on’ (dequenching) and ‘turn-off’ (quenching) need to be addressed with further research in the arena of OPC and IOPC.

## 4. Photonic Crystal Fibers (PCFs) for Fluorescence-Based Applications

### 4.1. PCFs: An Overview

**Figure 3:**
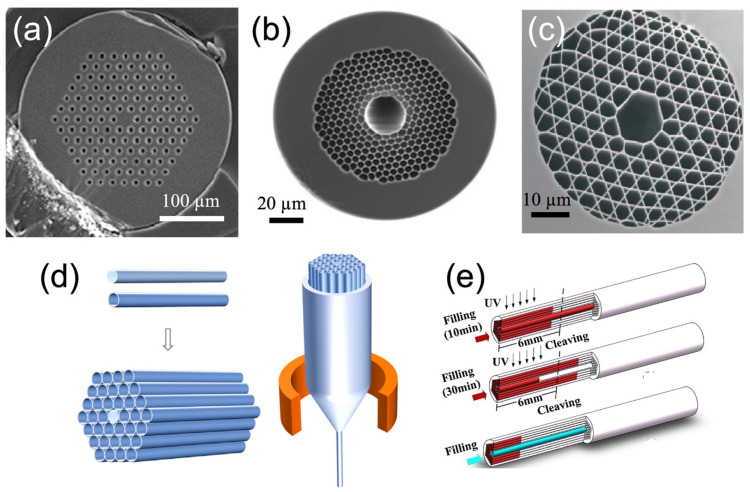
Design and fabrication of PCF. (**a**) A solid-core photonic crystal fiber, adapted with permission from [133]. (**b**) A hollow-core photonic crystal fiber (HC-PCF) with an expanded central channel suitable for admitting whole human cells, adapted with permission from [147]. (**c**) An alternative HC-PCF with the Kagome design pattern, optimized for gas-phase detection, adapted with permission from [148]. (**d**) A schematic for the thermal pulling of a preform into a thin fiber with the same structure, adapted with permission from [142]. (**e**) A schematic of the multiple cleavage method for selective filling of the hollow core with a fluorescent species, adapted with permission from [145].

Optical fibers are a versatile technology with many advantages that have led to their adoption in various sensing applications. Their ability to flexibly guide light using total internal reflection (TIR) makes them convenient for portable and hand-held sensing probes, and they can be miniaturized to act as optical components in lab-on-a-chip modules [131]. More recently, the field of optofluidics has integrated the light path and the sample containment into the same structures, with hollow fibers that admit both guided radiation and a continuous flow of analyte, ideal for real-time monitoring [132]. These geometries have the benefits of confining analyte to a very small space with a high surface area-to-volume ratio, which allows for excellent diffusion kinetics [133] and the ability to use small sample volumes on the microliter or nanoliter range [134]. However, maintaining low loss and acceptable wavelength selectivity in transmission requires a more sophisticated design; when a core is filled with liquid, the refractive index contrast between the core and cladding is no longer suitable for TIR. In order to overcome this limitation, a wide variety of designs for Microstructured Optical Fibers (MOFs) which include Photonic Crystal Fibers (PCFs) that make use of the PBG phenomenon to constrain the light while maintaining a path for analyte within the fiber have been incorporated. The cross-section of these fibers has the characteristic periodic refractive index that serves to create the PBG (see Figure 3a–c for some examples). These have been reported in an array of geometries for guidance of light from UV [135] to near-IR light [136] through media such as gases [137], liquids [138], and hydrogels [139]. While there are many sensing modalities that benefit from these fibers [140], in this review, we will focus on fluorescence detection and enhancement.

### 4.2. PCFs: History and Functionalization Methods

While PCFs may be obtained commercially, it is still informative to know the processes by which they can be engineered for particular purposes. The 1996 paper by Russell et al. describes a three-stage successive thermal pulling method [141]. A hexagonal silica column with a drilled hole was drawn into a thin fiber of 0.8 mm; by stacking pieces of this column into a periodic lattice and drawing the resulting stack on a thermal puller twice more, a PCF with a diameter of 38 µm was obtained [141]. Recent reports such as Li (2023) [142] describe a qualitatively similar process which can be used for a wide variety of photonic fiber and microstructured geometries (Figure 3). In on-chip applications, supported fibers printed or constructed using 3D lithography may also be effective photonic waveguides [143]. Other papers focus on the methods for functionalizing a channel so that capture molecules or reactive films may be deposited. This is not trivial, as PCF (with some exceptions [144]) consist of multiple hollow channels with a tuned refractive index and diameter. Hence, coating surfaces indiscriminately often results in a lossy fiber. Ensuring that only the desired channels are exposed to the functionalizing process requires a method of capping and selectively unsealing the cross-section and makes use of the fact that the central analyte channel is of a larger diameter than those in the outer ring of photonic cladding. A creative method using UV-curable adhesive has been demonstrated by Yang (2020) (Figure 3e) [145]. Beyond those considerations, various chemistries suitable for functionalizing glass or polymers (typical PCF materials) are employed [146].

### 4.3. PCFs: Relevance in Fluorescence Explorations

**Figure 4:**
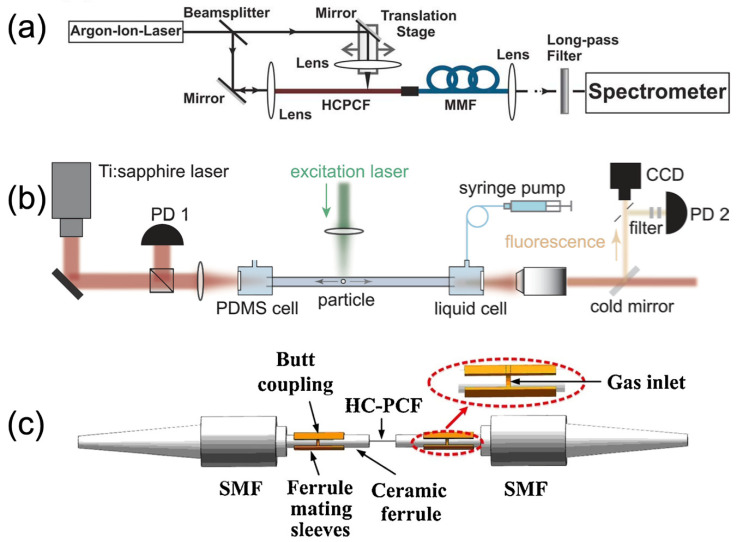
Detection instrument schematics for fluorescent detection. (**a**) A system that uses both end-on and perpendicular excitation to quantify the effects of reabsorption and fiber properties on sensor performance, adapted with permission from [152]. (**b**) A system using a single fluorescent bead with perpendicular excitation and fluorescence emission guiding to determine radiation strength with high spatial resolution, adapted with permission from [138]. (**c**) A system designed for the continuous flow of gas through the hollow core for real-time sensing. Excitation and emission are both parallel to the fiber, adapted with permission from [145].

Because of the versatility of design, PCFs are used for fluorescence detection in various ways. Making use of the flexible nature of optical fibers with the low-loss properties of PC guiding, Ghenuche et al. (2012) utilized PCF both to couple excitation light to a remote sensor and collect the emitted photons, and report single-molecule resolution significantly more sensitively than probes using ordinary optical fibers [149]. Other fiber designs use the evanescent field produced at the borders of the waveguide to excite fluorescence [133]; even though the evanescent interaction is short-range and comparatively weak, the fibers facilitate a long path length for interaction and are viable for both fluorescent and other sensing modalities [142]. These methods are described in other reviews [131,132,140]. However, an alternative strategy for fluorescent measurement in PCF utilizes the ability to colocalize the analytes and the full intensity of the guided light within the fiber’s core. In a Hollow Core Photonic Crystal Fiber (HC-PCF), the bandgap structure is designed with an analyte-filled core in mind. The bulk of the guided light energy fills the hollow core and interacts with the sample there, freeing the analyte from any requirements of being attached to the surface and allowing a very low limit of detection for fluorescent species [150]. Designs that depend on immobilized coatings on the inner surface of the core can calculate the ideal thickness of the coating to maximize signal without sacrificing the photonic properties of the fiber as a result of refractive index changes (Figure 4) [145]. Other studies use this sensitivity to refractive index as a sensing mechanism in conjunction with fluorescence spectrometry [151].

For HC-PCF fluorescence detection, three major configurations are common, depending on the transmission characteristics of the fiber (Figure 4). As mentioned, some fibers carry both excitation and emission photons [145,150]. While this is convenient, significant filtering is required prior to the signal transduction at the interfacial sensing component to ensure that stray excitation photons do not obscure the signal from the desired fluorescence. In other systems, excitation light is sent along the fiber, and the fluorescence output is measured perpendicular to the fiber [153], which may be used to allow for multiplexing of the signal [133]. These take advantage of the high field strength of the coupled light in the central channel to maximize the excitation. In many cases, however, excitation light is applied perpendicular to the fiber and the small amount of fluorescent emission from the analyte in that region is guided to the sensor at the end of the fiber [134,135,138,144]. Any sensor which sends the output along the fiber has a potential drawback: if a long path length of fluorophores is involved, the phenomenon of reabsorption can reduce the fluorescence output artificially, as emitted photons are absorbed by molecules between the emitter and detector. Some systems are optimized to avoid this where possible, but others simply quantify the effect and take it into account when analyzing their output [150,152].

### 4.4. PCFs: Outlook and Prospects

In general, fluorescence applications are either optimized for spectrometry or low-photon count fluorescence detection. Simply detecting whether an analyte is present assumes a different set of constraints than measuring the output spectrum to gain additional information about the analytes. Some fiber designs are optimized to have low loss over a broad range of wavelengths [150], suitable for spectrophotometry. Others use fibers designed to exclude the transmission of the exciting light and only guide a narrow band of wavelengths [135]. Some fibers are optimized to use extremely small sample volumes on the order of 1 nL [134], while others maximize faint signals by having long path length interactions of 10 cm or more [5,21]. The latter can detect fluorescent species at attomolar concentrations or with 20 pM fluorescein, as detected by Williams et al. (2013) [150].

Fluorescence is a versatile detection method, and the analytes of interest are naturally wide-ranging. Heck et al. detected unlabeled proteins by autofluorescence in the UV regime [135]. Other protein detection strategies use antibody capture and a fluorescently labeled secondary antibody [153]. Small biological molecules were detected using an enzymatic process that created fluorescent NADH; multiplexing and enzymatic sensitivity allowed this detection to be quantitatively stereoselective [133]. These signal-on approaches are attractive for measuring trace amounts of a specific analyte. Other strategies, such as fluorescence quenching [145] or aggregation-induced fluorescence [134], leverage another property to detect the presence of explosive gas in the ppb range and protein in the nM range, respectively. While not all examples are optimized for real-time sensing, those incorporating a continuous flow of analytes have time resolution ranging from two minutes [145] to half an hour [135], depending on the equilibration kinetics. This is an area that demands active research and development of fiber sensors to leverage their advantages in portable sensing. Regardless, it is clear that the design of PCFs for fluorescent analysis is a space with many dimensions for optimization and many potential applications in diverse sample compositions.

## 5. Applications of PC Cavity and Defect Modes in Modulating Fluorescence

The standard PC structure features many appealing optical properties, such as guided-mode resonance, photonic band gap, and near-zero index. When extra optical confinement is applied or a certain defect is introduced, a PC is capable of providing even more intriguing functionalities such as defects, band edges, and cavity modes. These configurations greatly enhance the fluorescence signal intensity by elevated quality factor, the optimal local density of states, confined mode volume, and enhanced spontaneous emissions. Such explorations have been found to be promising in fabricating defect-mode micro laser and fluorescent sensors.

**Figure 5:**
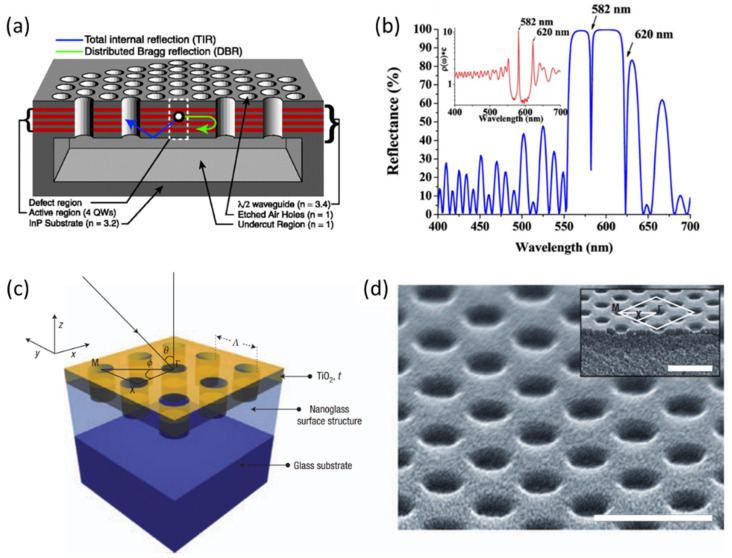
(**a**) Cross section through the middle of the PC microcavity. A defect is formed in the 2D PC by removing a single hole. The combination of Bragg reflection from the 2D PC and TIR from the low-index cladding (air) results in a three-dimensional confined optical mode, adapted with permission from [154]. (**b**) The calculated reflectance spectrum of the defect-mode 1DPC, glass-(PMMA-TiO2)15-(DCM/PMMA)-(TiO2-PMMA)15-air, at normal incidence of light by transfer matrix method. Arrows indicate the localized defect modes. The inset shows the calculated density of states of photons, ρ(ω)≡dk/dω, of the defect-mode 1D PC, in which the y-axis shows normalized ρ(ω) with respect to the value in vacuum ρ(ω)vac = 1/c, adapted with permission from [156]. (**c**) The layout of the two-dimensional PC device. (**d**) Scanning electron microscope images of a sample fabricated by the nanoreplica molding approach. Γ, X, and M are points of high symmetry. Λ is the period of the square lattice, t is the thickness of the high-index layer, and θ is the angle the incident beam makes with the vertical. Scale bars = 500 nm, adapted with permission from [37].

One of the pioneering works was introduced by A. Scherer et al. in 1999 [154]. They formed a laser cavity from a single defect introduced in a two-dimensional PC as shown in Figure 5a. Their optical microcavity consisted of a half-wavelength-thick waveguide as vertical confinement and a two-dimensional PC mirror as lateral localization. A defect in the PC was introduced to trap photons inside a volume of 2.5 cubic half-wavelengths (approximately 0.03 cubic micrometers). The laser was fabricated based on indium gallium arsenic phosphide material, and optical gain was provided by strained quantum wells designed for a peak emission wavelength of 1.55 μm at room temperature. The motivation for applying a PC lay in the inherent flexibility in geometry which allows fine-tuning of the defect-mode radiation pattern as well as the emission wavelength. The compact size and high spontaneous emission coupling factor of the defect microcavity also made it interesting as a low-noise, low-threshold light source. They observed pulsed lasing at 1.5 μm from optically pumped devices; however, the sample had to be cooled to 143 K and pumped with 10 nanosecond pulses. This work took advantage of simultaneously presenting wavelength-size small mode volume *V* and high-quality factor *Q*. Following this idea, Han-Youl et al. demonstrated square-lattice photonic band-gap lasers at room temperature from single-cell PC slab microcavities fabricated in InGaAsP quantum-well materials emitting at 1.5 μm [155].

According to these works, researchers recognized that the modification of the spontaneous-emission played a vital role in applications such as low-threshold lasers, high-efficiency light emitters, and single-photon sources. H. Y. Ryu et al. reported their work on enhancing spontaneous emission from the defect modes of a PC slab cavity [157]. They implemented finite-difference time-domain calculations and investigated spontaneous-emission enhancement from the monopole and the dipole modes of a hexagonal lattice cavity, with the consideration of the effects of the finite emitter linewidth and spectral detuning. They observed a large spontaneous-emission enhancement of >50 from the high-quality-factor monopole mode when the emitter linewidth was comparable with the resonant-mode linewidth. Meanwhile, in the case where broad-linewidth material was used, and a detuning effect was included, the dipole mode with a low-quality factor and a smaller mode volume was more advantageous for spontaneous-emission enhancement. Applying this similar idea and combining it with the inhibited density of states of photons within the stop band, Thomas et al. proposed a mirrorless lasing structure that consists of alternating layers of titania nanoparticles and polymethylmethacrylate PMMA with an active emission layer of organic dyes in PMMA [156]. According to Fermi’s golden rule, the rate of spontaneous emission at a frequency ω is proportional to the density of states ρ(ω). Therefore, if the gain medium is within a photonic band gap structure, the spontaneous emission rate at a particular wavelength can be enhanced or suppressed by a factor proportional to ρ(ω). They analytically calculated the density of states for the defect-mode finite 1D photonic band gap structure and plotted the normalized ρ(ω) with respect to the density of states in vacuum (1/c, where c is the speed of light in vacuum) shown in Figure 5b. The density of states had very low values within the photonic band gap, except at the localized defect modes, where the rate of spontaneous emission was enhanced by a large factor.

A significant amount of research has focused on the benefits of the stopband for fluorescence enhancement, but the extent of amplification is still limited [158,159]. In 2007, Ganesh et al. demonstrated a remarkable enhancement of fluorescence (ca. 108-fold) from quantum dots (QD) placed on two-dimensional PC slabs (shown in Figure 5c,d) with dual band gaps, which benefited from a combination of high-intensity near fields and strong coherent scattering effects of guided-mode resonance [37]. They fabricated two-dimensional PC slabs that operated at visible wavelengths and engineered their leaky modes so that they overlapped with the absorption and emission wavelengths of the quantum dots. After this work, they further presented a PC with a TM-polarized resonance at the cyanine-5 excitation wavelength and a TE-polarized resonance spectrally overlapping the fluorophore’s emission spectrum [160]. The former resonance increases the excitation of the fluorophore through enhanced electric field intensities, while the latter resonance redirects a proportion of emitted light toward the detection instrumentation [161]. Spots of cyanine-5-conjugated streptavidin on the PC demonstrate a 60-fold increase in fluorescence intensity and a 42-fold increase in signal-to-noise ratio relative to a glass slide.

**Figure 6:**
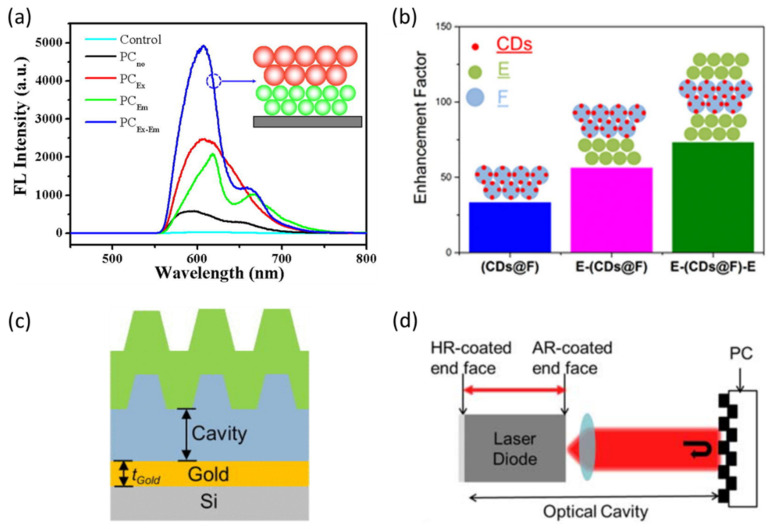
(**a**) Fluorescence spectra of Nile Red absorbed on the control sample and PCs with colloidal particle diameters of 180 nm (PCno), 210 nm (PCEx), 260 nm (PCEm), and 210down–260up nm (PCEx–Em). The inset shows the heterostructure PC films made of different self-assembly concentrations of P(St–MMA–AA) spheres, adapted with permission from [162]. (**b**) Fluorescence enhancement factor for all samples, measured as the fluorescence intensity ratio between the sample and the control, adapted with permission from [163]. (**c**) Cross-sectional schematic (not to scale) of the cavity-coupled PC, adapted with permission from [35]. (**d**) The resonantly reflected laser wavelength from the PC provides feedback to the diode. The cavity then lases at the resonant wavelength of the PC, adapted with permission from [79].

Heng et al. developed a facile approach on self-assembled heterostructure colloidal PCs with dual stopbands to enhance the fluorescent signal [162]. In their work, the intensity of fluorescent medium on heterostructure PCs can be up to 162-fold enhancement in comparison to that on the control sample, as shown in Figure 6a. Ehsan E. et al. reported self-assembled colloidal PCs constructed with a top and a bottom layer whose periodicity overlaps the excitation wavelength (E) of the emitters and a middle layer with a periodicity matching the fluorescence wavelength (F) whose thickness supports constructive interference for the excitation wavelength [161]. Their E-F-E double heterostructure renders an additional 5-fold enhancement to the extraordinary fluorescence amplification of Rhodamine B in monolithic E colloidal PCs and 4.3-fold acceleration of emission dynamics. They proposed an ion sensing platform by confining the fluorescent-sensing agent in the middle F layer. In doing so, the top and bottom E layers functioned as protective membranes to the middle F layer. They illustrated this idea by replacing the RhB with carbon quantum dots (CDs) that can be used as an Hg^2+^ ion sensing agent, as shown in Figure 6b. The 73-fold FL enhancement was observed for E-(CDs@F)-E, whereas a 56-fold enhancement for E-(CDs@F) heterostructure and 33-fold enhancement for monolithic (CDs@F) was observed. Another group presented a similar sandwich structure but replaced the E layer with a multilayer thin film so as to form a PC heterostructure cavity [33]. They found that the enhancement and spectral narrowing in dye emission can only be found within the overlap of the defect cavity mode of a multilayer stack with the band edge of the PC. The other defect modes, which did not overlap with the band edge, cannot show this effect. Most importantly, the defect mode that showed spectral narrowing was not the one residing at the peak of dye emission, as this was a mark of photonic lasing and not random lasing.

Fluorescence enhancement was also observed when the PC was combined with the external cavity. Compared to heterostructure, external cavities are easier to configure, and the requirement of sample fabrication is much more relaxed. In 2013, as Figure 6c shows, Cunningham’s group reported an approach to enhance fluorescence in which a compact gold mirror was placed underneath the PC so as to form a Fabry–Perot-type cavity. This configuration leads to a 6× increase in the signal-to-noise ratio of a dye-labeled polypeptide compared to ordinary PC-enhanced fluorescence [35]. The following work proposed a configuration where surface-adsorbed fluorophore-tagged biomolecules were excited on a PC surface that functioned as a narrow bandwidth and tunable mirror of an external cavity laser, shown in Figure 6d [79]. Here, they demonstrated ~105× improvement in the limit of detection of a fluorophore-tagged protein compared to its detection on an unpatterned glass substrate. As more novel structures are explored and more fabrication tools are available, PC defect or cavity-mode-enhanced fluorescence shows promise as a method for further increasing the signal-to-noise ratios obtainable for a wide variety of surface-based fluorescence assays.

## 6. Advancements in Fluorescence-Based Bioassays: Utility of Myriad PCs

PCs have been successfully used for various applications in the life sciences in a variety of methods, ranging from analytical tools for the study of molecular biology to the detection and study of numerous analytes of interest in medicine, healthcare, and diagnostics. Applications for PCs include chemical, gas, and drug detection, liquid biopsy methods for targets of interest in disease and therapeutics, and tools for studying cells and small molecule interactions, which have been described in greater detail in other reviews [163,164,165]. Fluorescence methods are currently one of the most applied tools in the study of biology and biosensors, with innumerable new methods for detection of small molecule interactions, microscopy, and sensitive quantitation gaining wide-spread use [166,167]. When the light-guiding properties of PCs are paired with current fluorescence techniques, enhancing the detectable signal of emitted photons, the combination creates a powerful tool for a wide range of applications in precision medicine and biology.

The versatility of PCs resonances and light-guiding properties, depending on design, allows PCs to be customized to fit existing fluorescence technologies, while greatly increasing resolution and sensitivity. Fluorescence biosensors typically employ labeling methods such as fluorophore or QD tags, where the fluorescent intensity increases with the presence of the analyte of interest [168]. However, other methods such as Förster Resonance Energy Transfer (FRET), where the close proximity of an emission donor and excitation acceptor causes the detected fluorescence wavelength to shift, have also been successfully applied to PC-integrated optical biosensors, using systems such as paired fluorophores or quantum dots (QDs) with a quenching structure like a gold nanoparticle (AuNP). One method presented by Zhang et al. (2017) used the FRET between QDs and AuNPs, amplified by a porous silicon PC Bragg reflector, to detect 16S rRNA sequences of actinobacteria through complementary [169]. Another approach described by Hu et al. (2020) utilized FRET between rare earth-doped upconversion nanoparticles and a polymethyl methacrylate opal PC bilayer to detect a prostate-specific antigen, an important marker in prostate cancer screening, by using a sandwich antibody method with a detection limit of 0.1 ng/mL and a clinically relevant dynamic range [170]. These FRET-based methods could also offer additional advantages for the study of small molecule and protein interactions.

**Figure 7:**
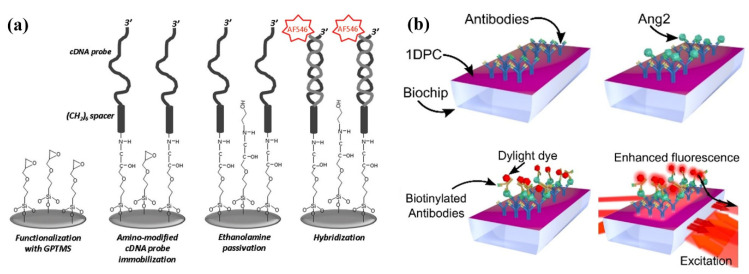
Fluorescence-based one-dimensional (1D) PC-sensing modalities for various biological analytes of interest. (**a**) A 1DPC biochip used to fluorescently detect microRNA, adapted with permission from [178] (**b**) 1DPC biosensor for Angiopoetin-2 tumor biomarker detection, adapted with permission from [173].

Fluorescence-based PC biosensors have been used to detect a wide variety of analytes of interest in diagnostics, medicine, and healthcare. One dimensional PCs (1DPCs) that rely on Bloch surface wave enhancement of fluorescence have been utilized for the detection of nucleic acids such as microRNA by Pileri et al. (2022), shown in Figure 7a [171]. The 1DPC is functionalized with complimentary single-stranded DNA probes that hybridize with a target microRNA, then a detection sequence with Alexa Fluor 647 dye can selectively bind to the surface, achieving a limit of detection of 32 ng/mL [171]. These 1DPCs biosensors have also been used by Frascella et al. (2013) to fluorescently detect the covalent binding of protein G [172] and in the detection of tumor biomarker angiopoietin 2 (Ang2) by Sinibaldi et al. (2015) and Rizzo et al. (2018), as shown in Figure 7b [173,174]. The fluorescent sandwich assay used on the PC biochip demonstrated a detection limit for Ang2 of 2.5 ng/mL in just 30 min. It showed good agreement with ELISA assays when tested with human plasma. The 1DPCs were also later employed by Occhicone et al. (2021) for the quantitation of Interleukin (IL)-10 with a detection limit of 110 pg/mL [175]. This effect can additionally be used to study smaller phenomena—Sepe et al. (2019) used the anisotropy of fluorophores on 1DPCs to examine the rotational diffusion kinetics of proteins bound to the surface [176]. PCs have also been used to amplify the fluorescent signal of more complicated sensing modalities, such as the CRISPR/Cas12a-mediated DNA walker that Li et al. (2022) reported for the ultrasensitive amplified detection of microRNA with an LOD of 4.1 fM. They also used an aptamer-modified approach in the same method to show the detection of a cancer-indicated protein carcinoembryonic antigen (CEA), achieving a LOD of 0.32 pg/mL, thus showing much greater sensitivity than ELISA and other fluorescence-based sensing methods [177].

**Figure 8:**
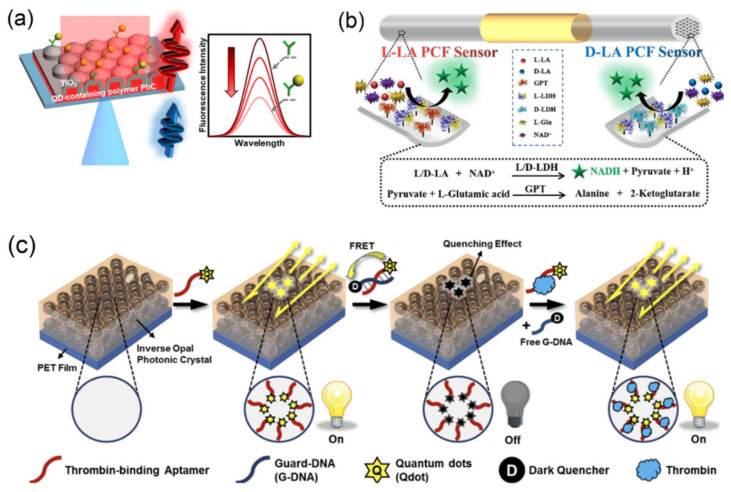
Embedded PC fluorescent biosensors. (**a**) Quantum dots encapsulated in a titanium dioxide PC for the detection of protein Troponin T, adapted with permission from [179]. (**b**) PC fiber for fluorescent detection of lactic acid enantiomers, adapted with permission from [133]. (**c**) Fluorescent aptamer detection of thrombin using a three-dimensional inverse opal PC, adapted with permission from [180].

There are also a variety of biosensing methods that have utilized fluorescence with more complicated PC structures, including two- and three-dimensional PCs, PC fibers, and embedded PCs to detect biological analytes. Ueda et al. (2022) describes a hybrid titanium dioxide PC embedded with QDs, as shown in Figure 8a, which enhances the QD fluorescence by matching the PC hybrid reflection spectra. The system was demonstrated through the detection of troponin T, a myocardial protein biomarker, using a capture antibody; this achieved a limit of detection of 58.2 fg/mL [179]. PC fibers, described in detail in previous sections of this review, have been used to detect proteins such as unlabeled BSA and alpha-fetoprotein, which achieved a dynamic range of 0.1–150 ng/mL [135,153]. Chi et al. (2022) demonstrated a PCF system for the detection of lactic acid enantiomers by using dual enzymes to detect both L-LA and D-LA enantiomers, as shown in Figure 8b. In the presence of the targets, the enzymes catalyze the production of NADH, which is then measured using laser-induced fluorescence (LIF) [133]. Another approach, demonstrated by Wang et al. (2016), paired a three-dimensional nanoporous PC with aptamer-conjugated quantum dots for the fluorescent detection of thrombin, shown in Figure 8c. The QD-aptamer complex cycles through an “on-off” state using a FRET effect between the complex and a quenching guard DNA strand, which is removed in the presence of the target thrombin. Emission light guidance from the 3D PC allowed for the ultrasensitive detection of the protein, with a calculated LOD of 3.8 pM [180].

There are several novel PC structures used for biological applications, such as opals, membranes, or colloidal PC biosensors, as described in the literature. Lee, W et al. (2018) demonstrates a method to immobilize antibodies on a three-dimensional inverse opal for the detection of influenza A (H1N1) [181]. The approach was expanded by Lee, N et al. (2018) through quantum dot-aptamer beacons to allow H1N1 virus detection with a smartphone camera [159]. Inverse opal PCs have also been used as a film sensor for formaldehyde vapor, with results in under 20 s and good sensitivity [182]. One inverse opal biosensor for the detection of the protein CEA (Zhang, 2022) relied on electrochemiluminescence enhancement [183]. Hou et al. presented a fluorescent chiral PC membrane made from liquid crystals that acts as a fluorescent off–on switch, which was sensitive to small changes in humidity and the presence of small molecules, such as formaldehyde [184]. Colloidal PCs, or periodically arranged nanoparticles, have also been used as biosensors in several applications for protein detection [185] and PC barcodes for the multiplexed detection of different ovarian cancer biomarkers [186]. The variety of fluorescent tools combined with a multitude of options for PC design creates various opportunities for innovation and applications in biosensing.

## 7. Photonic Crystal-Coupled Emission (PCCE): Insights and Applications

In an attempt to address the ever-increasing requirement of improving the sensitivity of biosensing devices, different nanofabrication protocols and photo-plasmonic platforms have been developed in recent decades. By and large, the fields that involve coupling the plasmon resonances of the metallic NPs with the emissions from the radiating dipoles to achieve signal transduction and associated spectroscopic signals at a metal–dielectric interface have advanced into an independent research field known as ‘plasmon-enhanced spectroscopy’ [46,48,59]. It has been observed that the most appropriate method to enhance the performance of the biosensors that depend on fluorescence is to modulate the local environment of the light-emitting species. In 2004, Lakowicz and co-workers demonstrated an effective approach by interfacing the radiating dipoles over the metallic thin films to realize sharply directional and highly polarized emission characteristics, upon satisfying the appropriate phase-matching conditions [55,187,188,189]. Following this revelation, different nanomaterials constituting metals, dielectrics, and low-dimensional carbon substrates (0D, 1D, 2D) have been interfaced with the propagating plasmons of the metal thin film to enhance the performance of the analytical detection fostered by SPCE technology [20,61,190,191,192,193,194,195]. Fundamentally, from the SPCE technology development point of view, the approaches to realize unprecedented emission intensity from the radiating dipoles can be categorized into the following: (i) implementation of appropriate nanoengineering techniques to enhance the radiative decay and global quantum yield [64,88,196,197], (ii) designing the suitable optical components for collection of the emission such as conical mirrors and associated lenses [198], (iii) substituting the underlying metallic thin film with an alternative platform with augmented performance in biosensing modalities [15,58]. The first two objectives have been explored in the past decade and concomitantly different processes, biosensing frameworks, and applications have been realized with the SPCE platform [66,199,200,201,202,203,204,205]. In spite of the wide-ranging applications rendered by the SPCE technology, the far-reaching capability of the platform has been hindered on account of the inevitable Ohmic losses intrinsically sustained by the SPCE platform [206,207]. In this regard, in an attempt to accomplish the third objective, Bhaskar et al. developed a robust, lossless, and practical biosensing platform using a 1DPC with the potential to entirely replace the metallic thin films used in the SPCE platform. In this penultimate section, the major outcomes, modalities, and highlights of the hence developed PCCE platform are discussed [39,41,43,56].

### 7.1. Simulations: Understanding the BSWs and IOMs Sustained by the 1DPC

**Figure 9:**
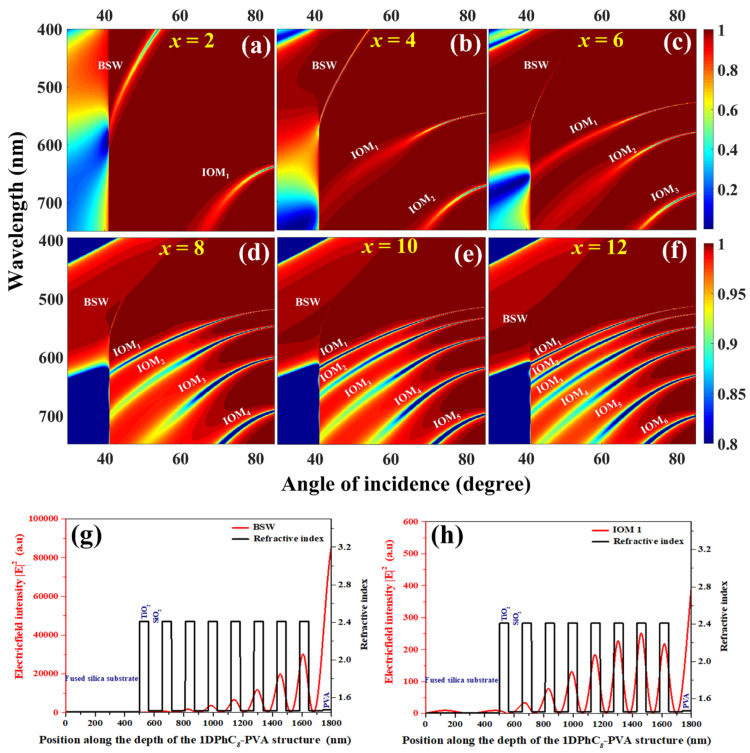
Dispersion diagrams for 1DPCx−PVA structures with (**a**) 2, (**b**) 4, (**c**) 6, (**d**) 8, (**e**) 10, and (**f**) 12 bilayers. The color bar represents the reflectance. The BSWs and IOMs are identified. (**g**,**h**) present the TE-polarized electric field intensity profiles for BSW and IOM supported by the 1DPC8-PVA structure. The corresponding refractive index profile of the 1DPC8-PVA structure is also shown. Adapted with permission from [56].

To begin, we present a brief outline of the observation and development of the different modes sustained by the 1DPC. In this regard, the results from the MATLAB simulations for the 1DPC are constructed on a fused silica substrate comprising a different number of bilayers; 2, 4, 8, 10, and 12, have been studied with the 60 nm-thick dielectric nanolayer over the 1DPC [56]. This dielectric is generally the layer in which the radiating dipoles are embedded while performing the experiments. Here, each bilayer consists of a pair of high (TiO_2_) and low (SiO_2_) refractive index nanolayers. This dielectric is given the characteristic property of the PVA polymer matrix as it is used in the subsequent PCCE experimental analysis. The 1DPCx−PVA (with x being the number of bilayers) was studied using the transfer matrix method (TMM) in the Kretchmann optical configuration; the different modes presented by the numerically calculated dispersion diagrams are presented in Figure 9 for TE polarization [56]. While the color bar represents the reflectivity of the entire structure, the vertical as well as horizontal axes indicate the wavelengths and the incident angles under consideration. It is important to note that the photonic band gap (PBG) is observed with a high percentage of reflectance below the critical angle (41.2°) and it shows a shift towards lower wavelengths with an increase in the angle of incidence. Further, from the close observation of Figure 9a–f, we see that the incident light is reflected for all the angles of incidence, excluding the specific regions of spectral domains where a significant drop in the reflectance is observed as BSWs and IOMs [43,56].

These interesting regions of the spectral domain are specifically categorized as BSWs and IOMs on account of their typical characteristic electric field intensities (EFIs), as observed in Figure 9g,h. We have shown only the representative EFI profiles for the 1DPC comprised of 8 bilayers of the HRI and LRI nanolayers, with a 60 nm dielectric (PVA) overcoat. While the BSWs generally occur very close to the critical angle, the IOMs appear at higher angles of incidence. Additionally, the strength (amplitude) of the BSWs is dramatically higher (Figure 9g) as compared to the strength of the IOMs (Figure 9h), as the former is the surface EM wave decaying into the 1DPC structure and the latter is a mode that is predominantly confined within the 1DPC (especially in the HRI domains) [41,56]. From a broad perspective, while the charge density oscillations of the metallic thin films dramatically impact the functionality of the radiating dipoles adjacent to it, the surface (BSWs) and bulk (IOMs) states of the 1DPC achieve a similar purpose in the case of the PCCE platform [41]. Interestingly, on account of the loss less nature of the PCCE platform, the all-dielectric platform performs exceedingly better as compared to the expected field enhancement at the metal–dielectric nanointerface in the SPCE platform. Such simulations assist the photonic experimentalists in designing appropriate fabrication protocols as per the need by considering the wavelength source and fluorophores utilized.

### 7.2. Experiments: Optical Framework, PCCE, and the Overlap with the Simulated Data

**Figure 10:**
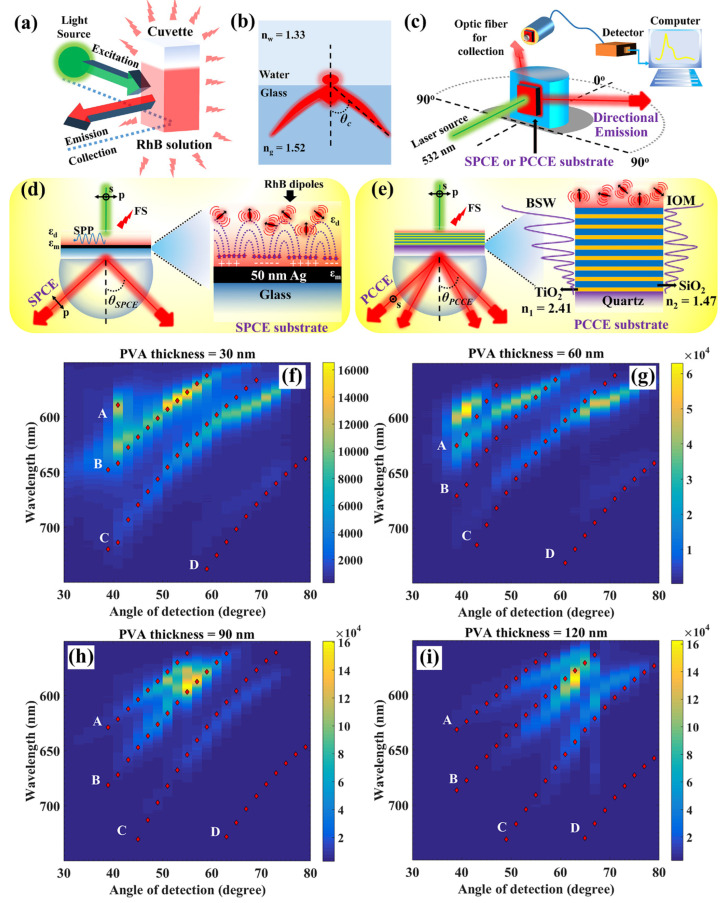
Conceptual schematic of (**a**) fluorescence emission (of RhB) recorded in conventional fluorescence spectrophotometer, (**b**) the angular dispersion of fluorescence emission as observed in the water–glass interface. The angle shown is the critical angle (θ_C_) of emission. (**c**) The optical setup used for SPCE experimental work with reverse Kretschmann (RK) configuration. Adapted with permission from [44]. Conceptual schematic of coupling of emission with (**d**) propagating surface plasmon polaritons (SPPs) of the metal thin film (with θ_SPCE_ as the angle of emission) and (**e**) surface-trapped modes of 1DPC (with θ_PCCE_ as the angle of emission). The magnified images on the right side of (**d**,**e**) display the coupling at the nanointerface. Adapted with permission from [44]. PCCE for the 1DPC-PVA-XX structure with XX (nm thickness) as (**f**) 30, (**g**) 60, (**h**) 90, and (**i**) 120 under 532 nm excitation. The color bar represents fluorescence intensity in arbitrary units. The red dots present the locations of BSW and IOMs from the numerically calculated dispersion diagram. Adapted with permission from [43].

Conventional fluorescence spectroscopy and related technologies where the measurements are generally carried out using the cuvette, as shown in Figure 10a, often suffer from several disadvantages, such as: (i) low signal collection efficiency on account of the detector being fixed at a particular angle (often at 90° to eliminate the light from direct irradiation from the laser source), (ii) less or moderate sensitivity for biosensing approaches, (iii) necessary need for using complex equipment, (iv) inadequate resolution in the emission peaks from the radiating dipoles, and (v) an isotropic emission attribute that results in significantly lower identification and signal collection from low quantum yield luminophores [44,46]. It has been observed that partially directional emissions can be realized from a radiating dipole placed at the interfacial junction of the glass–water interface (Figure 10b). In such cases, the emission in the relatively HRI (glass, n_g_ = 1.52) region is observed to be partly directional [44], on account of the effect of critical angle (θ_C_), as a result of which the evanescent field produced at the interface generates a non-isotropic (not highly directional) emission which is not polarized.

In light of these observations, SPCE and PCCE platforms have been developed to essentially realize highly directional emission with excellent control over the polarization of the emission observed. The optical setup utilized for carrying out the SPCE and PCCE experiments is shown in Figure 10c. Here, the SPCE and PCCE substrates comprise a 50 nm-thin Ag film and 1DPC, respectively [41,44]. The photo-plasmonic coupling between the radiating dipoles and the substrates under consideration is pictorially presented in Figure 10d,e for SPCE and PCCE substrates. While the surface plasmon polaritons couple with the emission from the radiating dipoles in the former, the coupling occurs with the BSWs and IOMs in the latter (as shown in the magnified conceptual schematics for easy understanding).

The generation of the evanescent field is supported by the appropriate optical configurations and collection of emission at specific angles (where the phase-matching conditions are satisfied). In typical experimentation (Figure 10c), the SPCE and/or PCCE substrates are coated with the radiating dipoles (via spin coating or other coating techniques) and affixed to a hemicylindrical prism using an index-matching fluid (such as glycerol). By and large, in the SPCE or PCCE experimentation, the excitation is performed in reverse Kretschmann (RK) configuration where the laser light is incident on the sample surface from the free-space side, and the coupled emission (SPCE or PCCE) is collected using the appropriate optical filters and polarizers from the curved surface of the prism [41,44]. It is worth noting that the experiments may also be carried out in the Kretschmann–Raether (KR) configuration with excitation from the prism side and collection from the free-space side. However, incorporating such geometries for performing ELISA-based immunoassays is tedious, with the requirement for multiple light channelizing sources, lenses, and mirrors. Such complexity is not associated with RK configuration as a 98-well plate can be simultaneously excited using a single laser source as the excitation is from the sample side.

Furthermore, it is informative to discuss the robustness and functionality of the PCCE platform by correlating the expected dispersion diagrams (from MATLAB simulations) and the observed experimental data. A representative dispersion overlap diagram is presented in Figure 10f–i. Here, the shaded blue-green-yellowish regions present the intensity of the fluorescence emission (with blue to yellow being the lowest to highest, as shown in the color scale on the right). Additionally, the dotted regions are the data obtained from the transverse matrix method-based MATLAB simulations. Here, the experimental data correlate to the fluorescence emission intensity gathered from the PCCE substrates coated with different thicknesses of the dielectric (in this case, PVA polymer matrix is utilized to embed the radiating dipoles) and the experiments are carried out in RK configuration, 532 nm continuous wave laser source, 550 nm LWP filter [41,44]. The highlight of these PCCE experiments for coupling the emission from the radiating dipoles with the different modes of the 1DPC is the excellent overlap observed between the experimental and the simulated data [43]. Furthermore, a good agreement is observed between the expected strength of the simulated modes as well as the data obtained from the PCCE measurements. On account of such outstanding agreement observed between the experimental and simulated data, the PCCE platform justifies a promising platform for monitoring the fluorescence enhancements by effective nanoengineering strategies.. The augmented fluorescence enhancements presented with cutting-edge biosensing frameworks using the PCCE platform can be further extrapolated for the detection of several analytes of interest simultaneously by tapping different modes of the PCCE substrate, thereby enabling multiplexed sensing. Therefore, the PCCE biosensing technology is envisaged to develop as a cornerstone for different lab-on-chip sensing tools with potential utility in academia and industry.

## 8. Photonic Crystal Enhanced Fluorescence Emission (PCEF): Insights and Applications

### 8.1. Fluorescence Enhancement Methods

Recently, numerous studies have focused on the issue of improving the absorption of single fluorescent reporters, effectively collecting their photon emission and eliminating the off-focus background to enhance the signal-to-noise ratio (SNR). By implementing high NA oil-immersion objectives and EM-CCD cameras, Total Internal Reflection Fluorescence (TIRF) microscopy is capable of producing high SNR single-molecule images with evanescent-field excitation; however, this is costly [208,209]. Plasmonic nanostructures have proven to be effective at enhancing the electric field excitation strength and increasing the fluorescence intensity ~10^2^ folds, but they have drawbacks such as significant non-radiative decay and low photon directionality [210]. Early fluorescence enhancement methods using dielectric optical microcavities show moderate increases in the emission rate caused by the mismatch between the high-Q cavity resonances and the inhomogeneously broadened emissions [211,212,213]. These problems were recently addressed with plasmonic–dielectric hybrid nano-gap and dielectric nanowire-slabs which showed an enhancement of 10^3^ times. However, the sparse distribution of highly localized “hot spots”, sophisticated nanofabrication process, exact alignment between cavity and fluorophore all limited its application [214].

### 8.2. Photonic Crystal Enhanced Fluorescence

Previous research used a microscopy-based approach for fluorescence enhancement from a dielectric PC surface over extended surface areas to overcome these issues. A bulk Cy-5-conjugated streptavidin layer on a one-dimensional PC has been shown to increase fluorescence intensity by 60-fold [215] and even 360-fold with a gold mirror reflector via a Fabry–Perot-type cavity [35,97]. Connecting the PC leaky mode and fluorescence-guided extraction, a two-dimensional PCs can produce a 108× fluorescence amplification for a thin layer of quantum dots (QDs) [37]. Yan et al. recently demonstrated a multiple heterostructure PC with a super-wide stopband to achieve a 10^2^-fold increase in broadband fluorescence [216], which required a sophisticated layer-by-layer fabrication of self-assembled 2D colloidal crystal monolayers. Florescence has also been enhanced using three-dimensional PC structures. Song et al. spin-coated a Ru dye layer on 3D opal PCs made up of multilayers of PMMA spheres in order to achieve a 320-fold luminescence enhancement with dual-stopband configurations [161].

### 8.3. Theoretical Framework of Photonic Crystal Enhanced Fluorescence

Beyond previous reports of quantum dot (QD) enhancement by PC surfaces [37], Cunningham’s group recently created a newly designed PC nanostructured surface that can serve as a general-purpose macroscopic substrate for the fluorescence microscopy of QD tags, with a simple low-cost fabrication process and significantly improved enhancement factor up to 3000 folds [78]. Radiative engineering achieves much greater enhancement by precisely matching two quality factors of radiation (Q_r_) and non-radiation (Q_nr_). The radiation quality factor relates to the stored optical energy of photonic cavity while the non-radiation quality factors depend on absorption or inevitable loss caused by fabrication imperfections. To maximize the enhancement factor toward theoretical limits, Q-matching requirements (Q_r_ = Q_nr_) must be satisfied for two PC resonance modes (both pump mode and fluorescence mode). Compared with unpatterned surface and bulk absorption, the PC is intended to provide a resonant optical pump mode at the wavelength of laser illumination, generating an increased electromagnetic field for stimulating surface-attached QDs. The increased excitation mechanism allows for the significant absorption of pump energy by QDs near the PC surface and generates more photons with an enhanced excitation rate than QDs in contact with a glass surface. Meanwhile, in the far-field, QD emission light coupled with the PC fluorescent mode with sharp spectral features radiates at a higher extraction rate, thus allowing sensors to capture more photons. Moreover, the dielectric PC improves quantum efficiency via the Purcell effect, resulting in a shorter spontaneous emission lifetime. The photonic dispersion provided by the PC band diagram governs the highly directional photon angular distribution in free space. Through the strategic selection of a microscope objective lens, the directional emission mechanism can improve the overall photon collection efficiency from the PC surface. Furthermore, the presence of QDs on the PC has shown blinking suppression for QDs near the surface, in which the QD remains in its “on” state for a greater percentage of the time.

**Figure 11:**
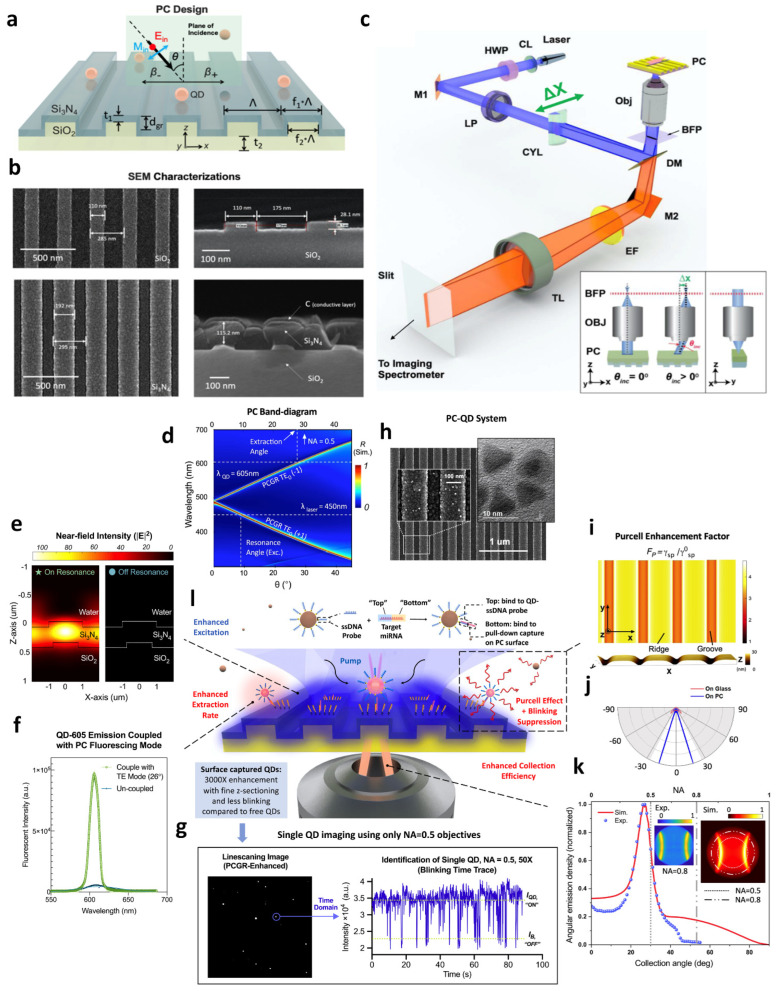
Photonic crystal enhanced fluorescence emission and blinking suppression. (**a**) Photonic crystal design. QDs are spread onto the PC surface, the excitation laser is TE-polarized with an incidence angle θ. Structure parameters of the PC: Λ = 295 nm, dgr = 28.1 nm, t_1_ = 115 nm, t_2_ = 1 μm, f_1_ = 0.651, f_2_ = 0.385; (**b**) SEM characterization of the PC-grating structure. The cross-section view and the bird’s-eye view before and after Si_3_N_4_ top grating deposition. (**c**) Photonic crystal enhanced fluorescence (PCEF) optical setup with line-scanning imaging system. Laser: laser diode (450 nm), CL: collimating lens, HWP: half-wave plate, LP: linear polarizer, CYL: cylindrical lens, DM: dramatic Mirror, OBJ: objective Lens, TL: tube lens, M_1_, M_2_: Mirrors, EF: emission filter (500 nm long-pass), BFP: back focal plane. (**d**) Simulation of angle-resolved PC reflection spectra (band-structure). (**e**) FDTD simulation of near-field intensity distribution of the PC cross-section, both on-resonance (θ = 9.2°, **left**) and off-resonance (θ = 20°, **right**). (**f**) Angle-resolved emission spectrum measurements for QD-605 when coupled with the enhanced fluorescent mode (green) and solitary on glass (blue), both with collection angle = 26°. (**g**) An example PCEF line-scanning image (**left**) is shown the surface-captured QDs experience 3000× enhancement compared to the free-floating uncaptured QDs. The strong enhancement factor enables single QD imaging using only NA = 0.5 objective lens. Time traces of diffraction-limited spot intensities (**right**) are used to identify single QDs by their distinctive two-level intensity distributions. (**h**) SEM image of the PC-QD coupled system with TEM image of the QD-605 as right inset. (**i**) Top panel: Numerical simulation for Purcell enhancement factor (Fp) 2D mapping as a function of position on the PC surface. A single dipole is used and assumed to be oriented in x-direction. Bottom panel: Topographic imaging for the PC surface using Atomic Force Microscope (AFM), x = 1.13 um, y = 0.4 um, z-height ranges from 0 nm (groove) to 28 nm (ridge). (**j**) Simulated far-field radiation pattern from the PC surface (blue curve) and glass (red curve). White regions represent angular regions that were collected by NA = 0.5. (**k**) Increased collection efficiency with BFP images and angular distribution of the emission. Theoretical (red) and measured (blue) angular emission intensity. (**l**). Schematic of the PC-QD system. Enhanced near-field excitation intensity, highly directional emission, enhanced extraction rate, and increased collection efficiency. Adapted with permission from [78].

The combination of several independent multiplicative effects that use a PC surface achieve an unprecedented 3000× enhancement in QD emission with blinking suppression, allowing the observation of individual QDs using a low NA objective while maintaining a high signal-to-noise ratio, a large field-of-view, and improvement of signal intermittency issues. The PC-QD system (Figure 11a,b) consists of QDs attached to the surface of a dielectric PC slab via biomolecular linkages. Figure 11h depicts a representative image of the PC-QD system captured by a scanning electron microscope (SEM). The PC slab enables photonic crystal-guided resonances (PCGRs) in a Si_3_N_4_ thin-film coating (n = 2.05) over a periodically modulated SiO_2_ substrate (n = 1.46). The surface is immersed in aqueous media and stimulated from behind with a transverse electric (TE)-polarized laser (laser = 450 nm) at an incidence angle. When the laser incidence angle satisfies the phase-matching criterion of the PC, it will be coupled as propagating guided modes via Bragg scattering. To examine the single-molecule detection capability and experimental enhance factors of the PC-enhanced fluorescence (PCEF) microscope, a line-scanning imaging system was built to examine PC under resonance conditions for a larger field-of-view (FOV). In Figure 11c, a half-wave plate rotates the major polarization of a collimated blue laser diode perpendicular to the PC grating after passing through a linear polarizer. The incident beam is focused by a cylindrical lens to a line profile and aligned with the rear focal plane (BFP) of the objective lens. The insert of Figure 11c explains how the incident angle can be accurately controlled by shifting the focus line on the BFP using Fourier transform. Line-scanning ability is accomplished by a motorized sample-moving stage, which moves perpendicular to the laser line direction. The incidence angle selection method derived from the phase-matching condition permits a direct comparison of the near-field distribution, excitation intensity, and QD-enhanced emission in a photonic resonator (PC-QD-linked mode) and an unpatterned substrate (solitary QD).

### 8.4. PCEF Platform Enables Single QD Digital Resolution

Excitation enhancement takes place when the PC supports a pump-resonance mode at the wavelength of the excitation laser, hence, boosting the local electric field (Figure 11e). The excitation light propagates inside the PC via the resonance-assisted transport pathway and forms ‘leaky eigenmodes’ that are confined along the PC’s top surface for a finite lifetime with the tunable Lorentzian line-shape resonance in the frequency domain. With continuous input energy from incident light, the PC serves as a photon energy-storing resonant optical cavity. Consequently, the local excitation field can be orders of magnitude greater than on an average unpatterned surface (Figure 11e), which can contribute to the increased absorption of surface-attached QDs. At resonance, a strong near-field intensity builds up close to the surface, and the resonance mode extends over the entire macroscopic surface via the evanescent field tails. Similar to TIRF setup, but with low NA lens, this delocalized characteristic has enabled the PC to interact with additional QD tags everywhere on its larger surface area within close proximity near the surface.

In the presence of Fano resonances, a significant alteration in the spectral density of states (SDOS) causes an increase in the extraction rate. When connected to a macroscopic nanostructure resonance, the spectral and angular emission of QDs can be drastically altered compared to their emission in free space. Sharp spectral features in QD’s fluorescence spectra are observed in Figure 11f, with enhanced differential radiated power. In addition, the near-field resonance has a direct correlation with its far-field features. In the far-field, the PCGR transport channel interferes constructively with direct background reflection, resulting in a reflection peak at the resonant wavelength. Upon examination of the PC band diagram reflection spectrum (Figure 11d), the PC is designed with a moderate quality factor of the pump mode when a laser incident at 9.2° corresponds to the QD excitation wavelength. Based on COMSOL simulation, the theoretical excitation and extraction enhancement factors are around 22× and 35×, respectively, for QDs of approximately 8 nm diameter.

### 8.5. Purcell Enhancement and Blinking Suppression

The spontaneous emission mechanism is also altered prior to the QD-emitted photons coupling with the PC extraction mode and “leaking out” to the far-field with a higher out-coupling rate. Due to the near-field excitation boost and greater density of states, the spontaneous emission rate of QDs placed on the surface of the PC is also increased. This Purcell effect enhances QD emission by modifying quantum efficiency (QE) without increasing the non-radiative decay rate due to the lossless nature of dielectric materials. The improved emission rate corresponds to the lateral position of the QD on the PC surface and is consistent over a broad wavelength range for single emitters. In comparison to a solitary QD, the local density of available photon states is higher in the QD-PC-coupled system. An excited-state QD will transition to the ground state with a rapid radiative decay rate and emit photons. The faster rate of this radiative decay process outperforms alternative nonradiative decay pathways where an electron is lost by Auger recombination or surface trapping, causing off-states, and hence, suppressing blinking. With the PC suppression effects, the percentage of on-time for QDs increased from 15% (on glass) to 87% (on PC). The power law distribution fitting curve also indicated a higher likelihood of “on” events and a lower probability of “off” events.

As a compelling application of the PCEF of QD tags, Cunningham’s group demonstrated an enzyme-free miRNA assay for a prostate cancer biomarker [217,218,219,220,221,222,223] in which each target miRNA molecule in the test sample was labeled with a QD tag affixed to the PC surface. The direct counting capability from PC-enhanced QDs enabled a miRNA detection limit of 10 aM and a dynamic range that extended to 1 nM.

### 8.6. Futuristic Scope and Oppurtunities

The experiments with PCCE and PCEF substrates can be further explored with different types of nano-engineering over these substrates, presenting newer opportunities for modulating the fluorescence signal intensity. While the metallic NPs suffer from inevitable Ohmic losses, it is important to carefully chose such nanomaterials by taking into consideration the different photonic modes sustained by the PCs [224,225,226,227]. In this regard, it is important to comprehensively evaluate the physics calculations pertaining to the photonic band gap sustained by different types of PC platforms [228,229,230]. Additionally, different dielectric NPs with LRI and HRI can be investigated to comprehend the effect of cavity hotspots that are generated over all-dielectric metasurfaces. Further, the hybrid photonic coupling between such NPs and the underlying PC can be tailored using different two-dimensional materials that sustain *π*-plasmons (for instance, graphene, WS_2,_ and MoS_2_). Moreover, an important outlook of these platforms emerged on account of its high surface stability (as compared to metallic thin films) that supports a wide variety of functionalization for the detection of different disease biomarkers. Adequate knowledge of the real and imaginary components of the nanomaterials used for building the nano-architectures over the PCs would assist in developing biosensing platforms with excellent performance.

Furthermore, the utility of fluorescence-based detection techniques (PCCE and PCEF) enables the experimentalists to integrate them with the smartphone-based detector systems. From a futuristic scope point of view, these fluorescence-based detection systems could be further examined for studying processes within the biological cell environment though appropriate tagging as well as using relevant up-conversion and down-conversion fluorescent moieties. Such explorations are envisaged to ameliorate the current biosensing modalities with up-to-date, rapid, and user-friendly early diagnostic tools empowered with portable, accurate, and sensitive hand-held devices. The novel nano-engineering methodologies over PCCE and PCEF platforms combined with the judicious synergy of state-of-the-art mobile-phone-based biosensing strategies would be amenable for resource-limited settings.

## 9. Conclusions

The current research efforts in the broad domain of nanotechnology and photonics are directed toward the development of effective strategies for achieving desired EM field intensities with versatile optical configurations, so as to facilitate enhanced sensitivity that in turn fosters the early detection of targeted analytes (ions, molecules, biomarkers, proteins, to name a few). Recently, PCs and their integrated investigations with fluorescence-based techniques are in vogue to fulfil these growing needs, and such research attempts have undoubtedly ameliorated the biosensor modalities. In this review, we presented a comprehensive discussion on the recent technological evolutions in the broad arena of PC-based fluorescence studies, thereby providing a focused overview of the latest developments in the subject. While this review provides an outline of the fundamentals of fluorescence and PCs to acquaint the inter-disciplinary researchers in the field, we also discuss the design principles and strategies of intriguing platforms such as opals, inverse opals, and PC fibers, as well as PCs sustaining defect, band edge, and cavity modes for effectively modulating the fluorescence signal intensity. Further, an all-inclusive discussion is presented with regard to the robust PCCE and PCEF technologies. In addition to this, the current challenges and futuristic scope of the latest methodologies pertaining to PC-enhanced fluorescence is exhaustively captured to facilitate the readers from the inter-disciplinary expertise such as physics, materials science, chemistry, and biosciences. This review is designed to enhance the current understanding of the field and is expected to guide and disseminate the strategies for the development of cutting-edge point-of-care diagnostic technologies in the communities’ encompassing academicians and industrial researchers.

## Data Availability

No new data were created or analyzed in this study. Data sharing is not applicable to this article.
